# Expression and Functional Activity of the Human Bitter Taste Receptor TAS2R38 in Human Placental Tissues and JEG-3 Cells

**DOI:** 10.3390/molecules21030306

**Published:** 2016-03-03

**Authors:** Ute Wölfle, Floriana A. Elsholz, Astrid Kersten, Birgit Haarhaus, Udo Schumacher, Christoph M. Schempp

**Affiliations:** 1 Department of Dermatology, University Medical Center, Freiburg 79104, Germany; birgit.haarhaus@uniklinik-freiburg.de (B.H.); christoph.schempp@uniklinik-freiburg.de (C.M.S.); 2 Department of Pharmacology, Biocenter, Goethe-University, Frankfurt 60438, Germany; Floriana.Elsholz@gmx.de; 3 Dermatohistological Laboratory Dr.Laaff, Freiburg 79111, Germany; astrid.kersten@posteo.de; 4 Department of Anatomy and Experimental Morphology, University Medical Center Hamburg-Eppendorf (UKE), Hamburg 20246, Germany; uschumacher@uke.de

**Keywords:** bitter taste receptor, calcium influx, placental tissue

## Abstract

Bitter taste receptors (TAS2Rs) are expressed in mucous epithelial cells of the tongue but also outside the gustatory system in epithelial cells of the colon, stomach and bladder, in the upper respiratory tract, in the cornified squamous epithelium of the skin as well as in airway smooth muscle cells, in the testis and in the brain. In the present work we addressed the question if bitter taste receptors might also be expressed in other epithelial tissues as well. By staining a tissue microarray with 45 tissue spots from healthy human donors with an antibody directed against the best characterized bitter taste receptor TAS2R38, we observed an unexpected strong TAS2R38 expression in the amniotic epithelium, syncytiotrophoblast and decidua cells of the human placenta. To analyze the functionality we first determined the TAS2R38 expression in the placental cell line JEG-3. Stimulation of these cells with diphenidol, a clinically used antiemetic agent that binds TAS2Rs including TAS2R38, demonstrated the functionality of the TAS2Rs by inducing calcium influx. Restriction enzyme based detection of the TAS2R38 gene allele identified JEG-3 cells as PTC (phenylthiocarbamide)-taster cell line. Calcium influx induced by PTC in JEG-3 cells could be inhibited with the recently described TAS2R38 inhibitor probenecid and proved the specificity of the TAS2R38 activation. The expression of TAS2R38 in human placental tissues points to further new functions and hitherto unknown endogenous ligands of TAS2Rs far beyond bitter tasting.

## 1. Introduction

Sweet and bitter taste receptors (TAS2Rs) can be summarized as highly conserved G protein-coupled receptors (GPCRs). These receptors are present in insects (*Drosophila*) as well as in vertebrates ranging from fish to man [1,2,3,4], although they possess structural differences. It is generally believed that the bitter taste of compounds prevents us from swallowing potential toxic substances. Sweet and umami tasting compounds, on the other hand, prompt us to eat food that contains carbohydrates and amino acids [5]. However many important and healthy nutrients also contain bitter compounds [6].

The bitter taste receptor TAS2R38 is especially well studied and mostly accountable for the human polymorphism in tasting phenylthiocarbamide (PTC). Previously it could be shown that for persons with the TAS2R38 taster variant, concentrations of PTC that are neutral for those with the non-taster form are extremely bitter [7,8,9]. The taster TAS2R38 protein and non-taster form differ mostly in the amino acid residues at position 49, 262 and 296. The functional allele of the receptor has a proline, alanine and valine (PAV), whereas the nonfunctional allele contains an alanine, valine and isoleucine (AVI) at these positions, respectively [8]. According to bioinformatics analyses, it has been proposed that the valine at the third position stabilizes the receptor structure and allows notable receptor activation only in tasters [10]. Furthermore individuals who are heterozygotes for the PAV/AVI allele expressed different amounts of “taster” PAV mRNA and the expression amount correlated with bitterness perception of PTC [11]. Balancing selection is probably responsible for the fact that both taster and non-taster TAS2R38 alleles are preserved in human populations. Bitter tasting to PTC and goitrin, the hydrolysis product of glucosinolates from cruciferous vegetable like broccoli, might persist because it could help iodine-starved populations to escape hyperthyroidism that worsen by goitrin [6]. Tishkoff and colleagues recently tested hundreds of individuals from 57 human populations in Central and West Africa with distinct diets (for example, Pygmy hunter-gatherers and Maasai pastoralists). Surprisingly, this study showed that local diet had no effect on the evolution of any of the PTC-sensitivity gene variants. This proves that TAS2R38 must have other important roles in physiological processes than just warning for toxins, otherwise these variants wouldn’t exist anymore [12]. Very recently it could be demonstrated that persons with the TAS2R38 taster variant get less bacterial infections, because the bacterial components acyl-homoserine lactones (AHLs) activate these bitter taste receptors in upper respiratory epithelial cells [13,14]. As a consequence the bacteria were either directly killed by NO production in this activated cells or the bacteria were removed by increased mucociliary clearance. However, human bitter taste receptors (TAS2Rs) are not only expressed in mucous epithelial cells of the tongue and upper respiratory tract but also in epithelial cells of the colon and stomach and in cornified epithelial cells of the skin [15,16,17]. To investigate if also other epithelial tissues express bitter taste receptors human tissue microarrays containing 45 tissues from healthy human donors were stained. We selected TAS2R38; the best characterized TAS2R as target. Unexpected high TAS2R38 expression was detected in the placenta. Stimulation of the placental cell line JEG-3 with diphenidol, a synthetic TAS2R ligand for 16 TAS2Rs, including TAS2R38, demonstrated the functionality of the TAS2Rs by inducing calcium influx after stimulation. Furthermore, JEG-3 cells contain the TAS2R38 taster variant. The TAS2R38 ligand PTC specifically induced calcium influx that could be inhibited by pre-incubation with the TAS2R38 antagonist probenecid (*p*-(dipropylsulfamoyl) benzoic acid) [18]. Probenecid inhibits the taste receptors TAS2R16, 38 and 43 through an allosteric reaction mechanism. Probenecid is a FDA-approved inhibitor of the Multidrug Resistance Protein1 (MRP1) transporter that is clinically used to treat gout in humans or is co-administered with antibiotics and chemotherapeutics to reduce their excretion.

## 2. Results

TAS2Rs were expressed in mucous epithelial cells of the tongue but also outside the gustatory system in epithelial cells of the colon and stomach, upper respiratory tract, in Purkinje cells of the cerebellum and in the cornified squamous epithelium of the skin [13,14,15,17,19]. In the present work we addressed the question if bitter taste receptors might also be expressed in other epithelial tissues as well.

**Figure 1:**
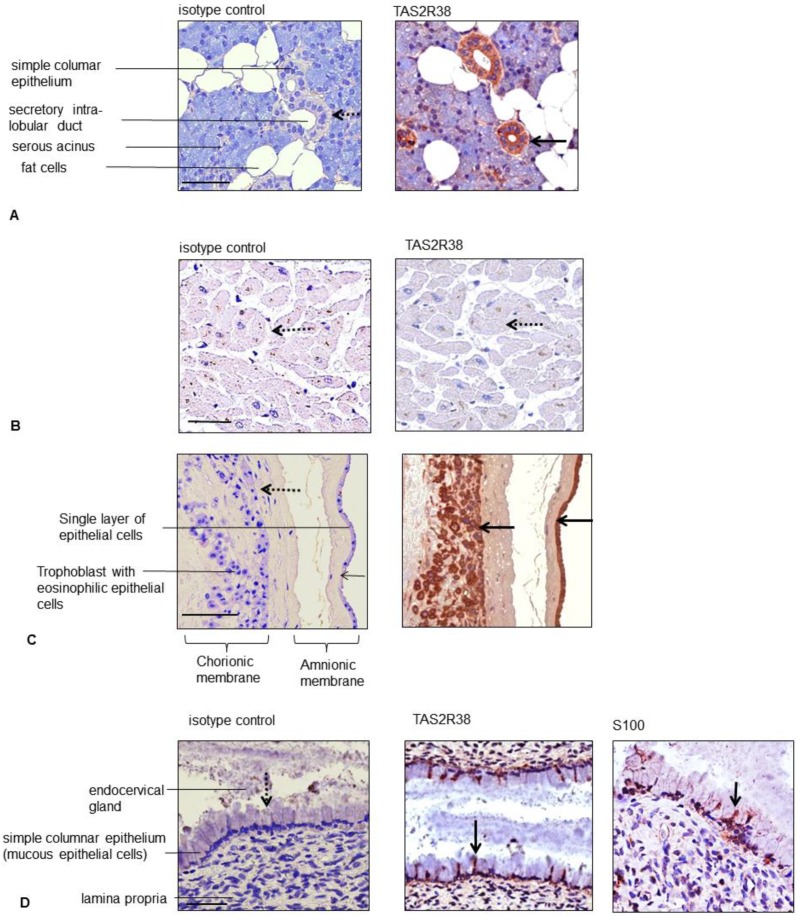
Staining of human tissues of the human tissue microarray against TAS2R38. Parotid gland (**A**); heart (**B**); human placenta (chorionic plate) (**C**) and endocervix (**D**) were stained with an isotype control antibody or an antibody against TAS2R38 and S100 (only the endocervix). Arrows in bold indicate positively and dashed arrows negatively stained cells; the bars correspond to 50 µm.

Although numerous commercial antibodies have become available for TAS2Rs, many of them do not appear specific [20]. Therefore we focused on the human TAS2R38 antiserum with proven specificity [21]. By staining a tissue microarray (TMA) with 45 tissue spots from healthy human donors with the best characterized bitter taste receptor TAS2R38 we observed apart from the already known positively stained tissues (e.g., gastrointestinal mucosa and ducts of the parotid gland, Figure 1A) and unstained tissues (lymphoid tissues such as lymph nodes, thymus, spleen as well as heart; Figure 1B) an unexpected strong TAS2R38 expression in the placenta. No staining was seen in the human placenta with an isotype control antibody. The placenta is a temporary organ required for the development of the embryo and fetus. It allows the exchange of metabolic products between the fetus and the mother. The placenta functions as a fetomaternal organ with two components: the fetal placenta (chorionic plate) and the maternal placenta (the basal plate). The fetal chorionic plate is composed of amnion, chorion and syncytiotrophoblast. The latter is an external layer that forms cords which invade the wall of the uterus to establish nutrient circulation between the embryo and the mother. The amnion epithelium (a membrane that surrounds and protects the embryo) and the syncytiotrophoblast showed a strong TAS2R38 staining (Figure 1C). The basal plate (maternal placenta), the part of the uterus to which the villi anchor, stained only weakly for TAS2R38 (data not shown). A few TAS2R38-positive cells could also be seen in mucous epithelial cells lining the endocervical glands (Figure 1D). For better characterization of these cells, S100 staining was performed to determine if the TAS2R38-positive cells were of myoepithelial or neuronal origin or dendritic cells. The S100-positive cells might be myoepithelial cells that are contractile and aid in secretion (Figure 1D). The stratified squamous epithelium of the exocervix was strongly positive for TAS2R38 expression. All staining results were summarized in Table 1.

### 2.1. Expression of TAS2R38 in the Placental Cell Line JEG-3

**Figure 2:**
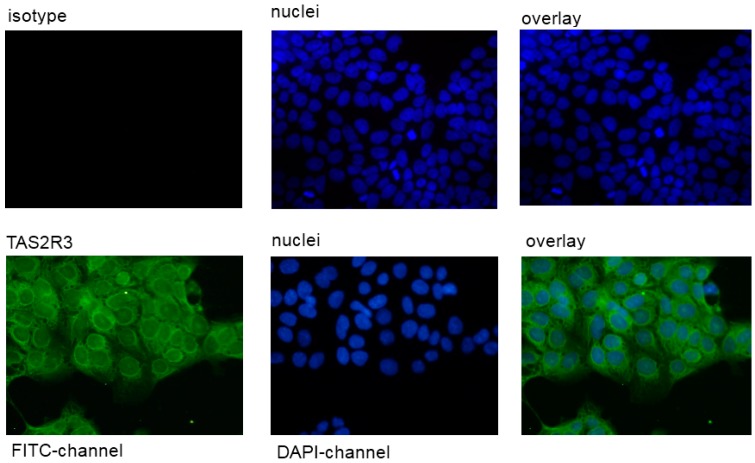
Staining of the human cell line JEG-3 against TAS2R38. The rabbit anti-TAS2R38 antibody was visualized by a secondary FITC-coupled antibody in the *FITC channel*. The isotype control showed no fluorescence signal in this cell line. The pictures were photographed at magnification of 400×. The bar corresponds to 50 µm.

It could be demonstrated that the placental JEG-cells expressed TAS2R38. No fluorescence signal was detectable with an isotype control antibody (Figure 2).

### 2.2. PTC Induced Calcium Influx in the Taster Cell Line JEG-3

**Figure 3:**
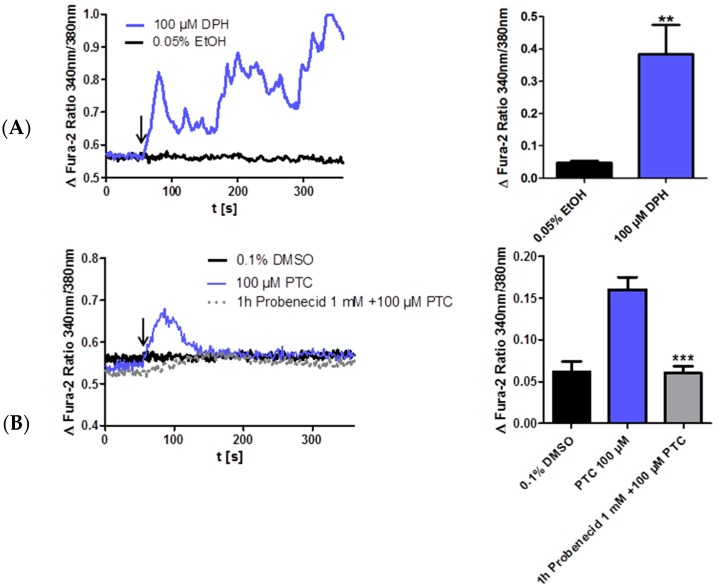
DPH and PTC induce calcium influx in JEG-3 cells. (**A**) JEG-3 cells are stimulated after 60 s with DPH (100 µM) or DMSO (0.1%) as indicated with the arrow and calcium currents are analyzed by calcium imaging. Calcium influx is expressed as the difference of the fura-2 ratio before and after stimulation; (**B**) JEG-3 cells are stimulated after 60 s with PTC (100 µM) or DMSO (0.1%) as indicated with the arrow. Calcium currents are analyzed by calcium imaging. Calcium influx is expressed as the difference of the fura-2 ratio before and after stimulation. 1 h pre-incubation with probenecid (1 mM) reduces PTC-induced calcium currents to solvent level. Data + SEM. *n* = 5. *t*-test, unpaired, two-tailed. ** *p* < 0.01, *** *p* < 0.001.

Next we determined if the TAS2R38 protein is also functional in JEG-3 cells by measuring intracellular calcium influx after stimulation with diphenidol (DPH). DPH is a synthetic TAS2R agonist which activates 16 TAS2Rs including TAS2R38 as described [22]. DPH medication has long been clinically developed as an antiemetic and an anti-vertigo agent [23]. According to data from the literature [17] JEG-cells were stimulated with 100 µM DPH and showed a significant calcium influx (Figure 3A). However, DPH is a muscarinic receptor antagonist and therefore not specific for TAS2Rs. This might be the reason why the calcium transient trace to DPH shows an atypical phasic curve. PTC is a specific TAS2R38 agonist in taster variants (PAV haplotype). Therefore we determined the TAS2R38 variant in the JEG-3 cell line by restriction enzyme analyses (see Experimental Section). The DNA of the placental cell line JEG-3 contains the taster variant (PAV) and could be used for PTC activation analyses. As control the DNA of the keratinocyte cell line HaCaT that is heterozygous (PAV/AVI) and the DNA of the human neuronal cell line SK-N-SH that contains the homozygous non-taster variant (AVI) were also analyzed and presented in the Experimental Section. Furthermore we confirmed the taster status of JEG-3 cells by sequencing the DNA of JEG-3 cells and SK-N-SH as non-taster cell line. The required PTC concentration for TAS2R38 activation was 100 µM according to data of the literature [9]. 100 µM PTC induced calcium influx in JEG-3 cells without showing cytotoxic effects (data not shown). To further demonstrate the specificity of the PTC activation in JEG-3 cells this activation was repressed with the TAS2R38 inhibitor probenecid as described by Greene and colleagues (Figure 3A,B). These results in JEG-3 cells demonstrate a specific TAS2R38 activation.

## 3. Discussion

Previously, Clark and colleagues have already speculated that TAS2Rs might also be expressed in other extra oral tissues apart from the respiratory and gastrointestinal endocrine cells [24,25]. Furthermore Dong and colleagues described that platypus, a non-placental mammalian, has one of the smallest TAS2R repertoires in mammals with only four TAS2R genes, which was suggested to be due to the non-bitter tasting semiaquatic diet (such as underwater crustaceans) [26]. Dong and colleagues postulated that herbivorous and omnivorous mammals would be expected to need a greater level of TAS2Rs compared to carnivores, because plants are much more likely to contain bitter tasting toxins than animals. The number of TAS2R genes increased almost five-fold in the placentals/marsupials mammalian lineages compared to monotremes. As several extra oral tissues strongly express TAS2Rs in humans, these receptors must have other biological functions than taste perception. By staining a tissue microarray with 45 human tissues unexpected high TAS2R38 expression was found in the syncytiotrophoblast. This multinucleated cell layer forms the barrier between the fetal and maternal circulation. It is therefore attractive to speculate that it confers specific information between the mother´s blood and the fetus. In addition, the amnion which forms the first protective layer around the embryo also showed a strong TAS2R expression. One known natural ligand of TAS2R38 is the acyl-homoserine lactone AHL-12, the quorum sensing compound from pseudomonas that was for example described in neutrophils [27]. So it might be possible that TAS2R38 in the placenta acts as a sensor for bacterial infection. The expressed TAS2Rs in the placenta are also functional, because the TAS2R38 agonist PTC induced calcium influx in the placental cell line JEG-3 that contains the TAS2R38 taster allele. Furthermore this calcium influx could be inhibited by the recently described TAS2R38 inhibitor probenecid. However probenecid is not a selective inhibitor for specific bitter taste receptors (TAS2R16, -38 and -43), it inhibits also organic anion transporters (OAT1) and is clinically approved for the treatment of gout [28].

**Figure 4:**
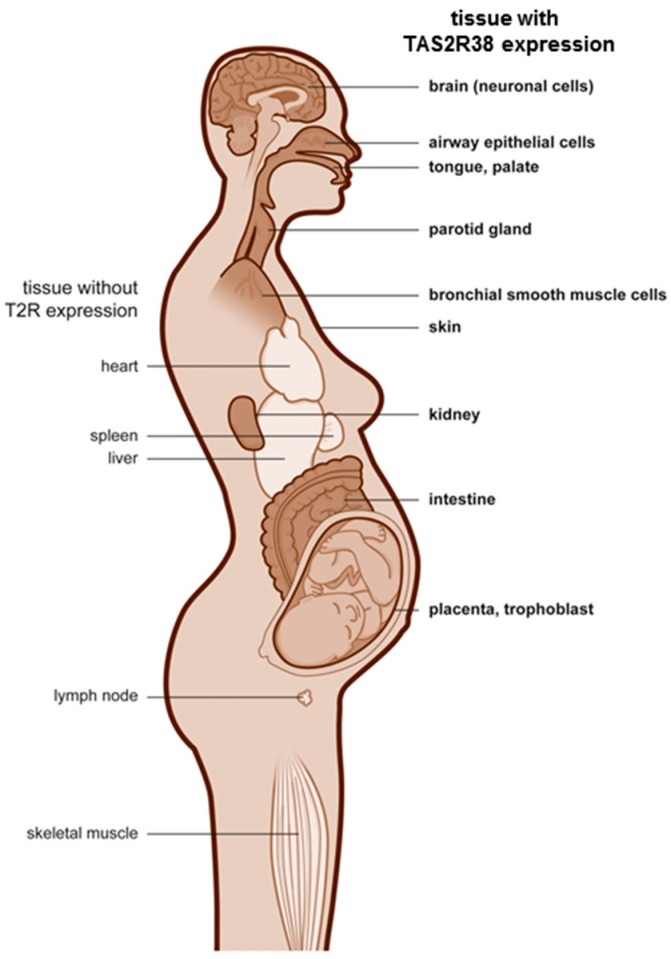
TAS2R38 expressing human tissues. The cartoon highlights organs that express TAS2R38 in the human organism in bold on the right site, and organs that are negative for TAS2R38 on the left site.

It can be speculated that structures that protect the embryo express TAS2R38 whereas some mesodermal structures such as the lymphatic system, blood, the heart, and skeletal muscle are negative. Furthermore TAS2R38 expressing cells in the placenta might be chemosensors which control hormone secretion in analogy to the enteroendocrine cells described in the gut [15]. The cartoon in Figure 4 highlights organs that express TAS2R38 in bold on the right site.

Our findings agree with a transmission electron microscopy study of Witt and Reutter who investigated the embryonic and fetal development of human taste buds. Their results suggested a dual function of embryonic/fetal taste buds, including non-gustatory, paracrine functions prior to the 14th week and gustatory after the 14th week [29]. Extra oral taste receptor expressing tissues might keep this first paracrine function.

Accumulating evidence indicates that not only taste receptors, but also odorant receptors are widely expressed throughout the human body beyond the orinasal cavity [20]. Therefore odorant and taste receptors should be simply considered as G protein-coupled receptors (GPCRs) as suggested by Foster and colleagues [20]. Hormones and bioactive substances can affect peripheral taste sensation [30]. Sweet taste responses can be modulated by cannabinoids [20,31], adenosine [32], leptin and glucagon-like peptide1 [30], whereas cholecystokinin is described as a regulator of bitter taste sensation [30]. Although these studies focused on olfactory or gustatory responses, they might be also relevant in other human tissues with taste GPCP expression.

## 4. Experimental Section

### 4.1. Antibodies and Reagents

The following antibodies and dilutions were used for immunohistochemical staining experiments: the polyclonal rabbit anti-human TAS2R38 antibody (Abcam, ab65509, Cambridge, UK), 1:1000; the polyclonal rabbit anti-human S100 antibody (Dako, Z0311, Glostrup, Denmark), 1:200 and the rabbit immunglobulin negative control (Dianova, DLN-13121, Hamburg, Germany, 1 mg/mL), 1:750. The secondary antibody multi-link-biotin, the streptavidin-horse-radish peroxidase (HRP)-label and the AEC-substrate were from Dako and were used according to the manufacturer’s instruction. The polyclonal swine anti-rabbit-FITC antibody was from Dako and used at a dilution of 1:30. The AccuMAx Array (Biocat, Heidelberg, Germany) contains 45 human tissues from various healthy organs with 2 spots of each case. The diameter of each spot on the slide was 1.0 mm. Each tissue section was extracted from various donor blocks and transferred into a ready-made recipient block. The donor blocks were formalin-fixed and paraffin-embedded. Probenecid, diphenidol hydrochlorid and PTC were obtained from Sigma-Aldrich (Taufkirchen, Germany) and the QIAamp DNA Blood Mini kit from Qiagen *(*Hilden, Germany).

### 4.2. Immunohistochemistry

Sections of the arrays were deparaffinized and subsequently subjected to a 20-min pretreatment in target retrieval solution (pH 6.0; Dako) at 100 °C or a proteinase K (Dako) treatment. Immunostaining was performed with the anti-human TAS2R38 antibody and the anti-S100 antibody. Application of the primary antibody (4 °C, overnight) was followed by incubation with biotinylated swine anti-goat, anti-mouse and anti-rabbit antibody immunoglobulins (1 h, RT), streptavidin conjugated to horseradish peroxidase (20 min, RT), AEC solution as chromogen and hematoxylin counterstaining. Stainings with the rabbit immunglobulin fraction served as isotype control. The tissue arrays were scanned with the MIRAX scanner (Zeiss, Jena, Germany) and the staining was evaluated according to anatomical structures.

### 4.3. Restriction Enzyme-Based Detection of the TAS2R38 Gene Allele to Distinguish between PTC Taster and Non-Taster

**Figure 5:**
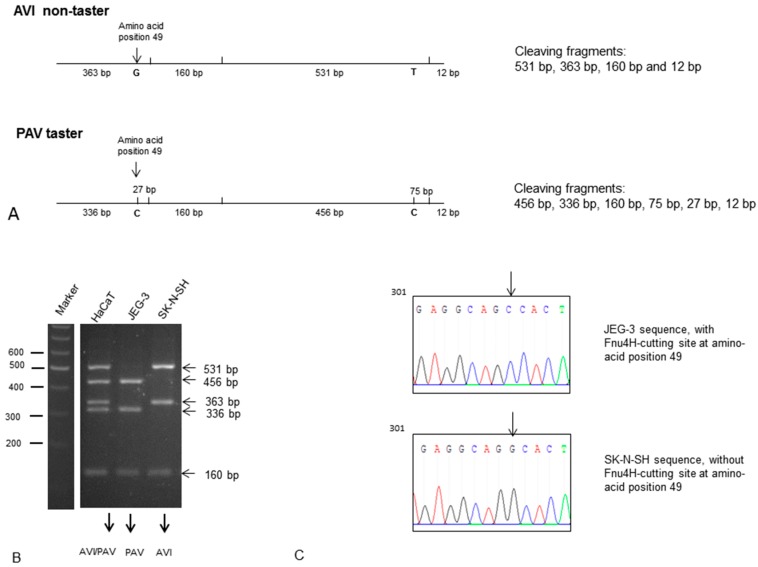
Determination of the TAS2R38 gene allele in the cell line JEG-3. (**A**) Scheme to demonstrate the cleaving products by a restriction enzyme-based analysis of PTC taster and non-taster. PTC tasters have the haplotype PAV and non-tasters have the haplotype AVI (A = Ala, V = Val, I = Ile) at the protein positions 49, 262 and 296; (**B**) Cleaving pattern of the JEG-3 cell line. (**C**) Gene sequence of JEG-3 DNA. Both methods identified JEG-3 as taster cell line according to data from the literature [8].

The most common single nucleotide polymorphisms (SNPs) in the TAS2R38 are in protein positions 49, 262 and 296. PTC tasters have the haplotype PAV (P = Pro, A = Ala, V = Val) and non-tasters have the haplotype AVI (A = Ala, V = Val, I = Ile). The sequence differences at the first and middle positions can be used to distinguish PAV tasters and AVI non-tasters by using a restriction enzyme that overlaps these two regions and so differentially cuts DNA depending upon the allele. The DNA for this reaction was isolated from cells with the QIAamp DNA Blood Mini kit according to the manufacturer’s instruction. First a 1067 bp fragment of the N-terminal portion of TAS2R38 is amplified by PCR (forward primer 5′catccctctaagtttcctgccaga, reverse primer 5′ttgggataatggcagcttgtccctc, annealing tem-perature: 58 °C*).* Then the PCR product was extracted from a 0.8% agarose gel with the gel extraction kit from Qiagen and then cut with Fnu4H (New England Biolabs GmbH, Frankfurt, Germany) at 36 °C for 15 min. Fnu4H cuts DNA at the sequence GCNGC (N is A, G, C or T). This sequence overlaps both polymorphism sites. At the amino acid position 262, PAV tasters contain the Fnu4H recognition sequence (G**C**TGC) while AVI non-tasters do not (G**T**TGC). At the amino acid position 49, PAV tasters contain also the Fnu4H cutting site (GCAG**C**) while AVI non-tasters do not (GCAG**G**). The restriction pattern of the TAS2R38 PCR product indicates the sequence at both polymorphic sites and was determined in a 2% agarose gel. A 531 bp Fnu4H fragment in non-tasters is cleaved to a 456 bp and a 75 bp fragment in PAV tasters. A 363 bp Fnu4H fragment in non-tasters is cleaved to a 336 bp and a 27 bp fragment in PAV tasters (see Figure 5A for a schematic description of the cleaving products). Heterozygosity displays both the cleaved and un-cleaved products. DNA of the JEG-3 cell line was screened for PTC taster and non-taster status (Figure 5B,C). Furthermore the JEG-3 DNA was sequenced using the GATC service (GATC Biotech AG, Konstanz, Germany). The stimulation of JEG-3 cells was performed with 100 µM PTC according to the literature [9].

### 4.4. Immunofluorescence

To perform TAS2R38 labeling, the cells were stained with the polyclonal rabbit anti-human TAS2R38 antibody. The required secondary antibody (swine anti-rabbit-FITC antibody) was applied for 2 h at RT according to the manufacturer's instructions. The cells were then stained with DAPI and mounted in fluorescence mounting medium (Dako). Images were taken with a fluorescence microscope (Zeiss) equipped with the Axiovision software.

### 4.5. Cell Culture

The human placental cell line JEG-3, the human keratinocyte cell line HaCaT and the neuronal cell line SK-N-SH were from CLS Cell Lines Service (Heidelberg, Germany) and cultured in Dulbecco’s modified essential medium (DMEM; Thermo Fisher Scientific GmbH, Schwerte, Germany) containing 10% fetal calf serum (FCS; PAA, Pasching, Austria) at 37 °C in a humidified atmosphere with 5% CO_2_. For fluorescence staining the cells were seeded in a four-field chamber slides (Thermo Fisher Scientific GmbH, 1 × 10^5^ cells/mL). For calcium imaging experiments, cells were seeded on polylysine (Sigma-Aldrich, Munich, Germany) coated cover slips at a density of 1.5–2 × 10^5^.

## 5. Fluorescence Measurements

Intracellular Ca^2+^ concentration measurements in single cells were carried out using the fluorescence indicator fura-2-am (Thermo Fisher Scientific GmbH). Cells were washed with a buffer containing 130 mM NaCl, 5 mM KCl, 10 mM HEPES, 10 mM Glucose and 1 mM CaCl_2_ adjusted to pH 7.4 and loaded with 2 µM fura-2-am and 0.04% Pluronic F-127 (Thermo Fisher Scientific GmbH) for 30 min. After rinsing twice, cells were allowed to de-esterify fura-2-am for 30 min. Measurements were performed with a monochromator-based imaging system attached to an inverted microscope (Axiovert S100, Zeiss). Fluorescence was excited at 340 and 380 nm and emission measured at 510 nm. After 60 s, cells were stimulated with diphenidol (DPH) or 100 µM PTC, the concentration tested in the absolute detection thresholds in the alternative forced-choice (2-AFC) trials or the respective solvent control. TAS2R38 receptor inhibition was performed by pre-incubation of JEG-3 cells for 1 h with the TAS2R38 antagonist probenecid (1 mM) before PTC stimulation. After correction of background fluorescence, the fluorescence ratio was calculated using Axiovision software.

## 6. Conclusions

We could show for the first time that the taste receptor TAS2R38 is expressed and functionally active in placental tissues, namely in the syncytiotrophoblast and in the amnion both of which protect the embryo. Therefore, apart from the prevention of toxic food intake, TAS2Rs might play a general role in the communication with environmental factors and the protection of the body against the environment.

## Figures and Tables

**Table 1:** Summary of TAS2R38-positive and -negative human tissues.

Blastodermic Layer	Organ System	Cells/Tissues	TAS2R38 Expression
ectodermal	central nervous system	Purkinje cells (Golgi apparatus like staining pattern) in cerebellum	+
		neurons/glial cells in the spinal cord	-
ectodermal	skin	keratinocytes, fibroblasts	+
		mucous membrane (tongue, palate)	+
ectodermal	placenta	amnion epithelium,	+
		syncytiotrophoblast, decidua cells	+
ectodermal	gastrointestinal mucosa and ducts	mucous epithelial cells and gland ductus of:	+
		ileum, cecum, colon, rectum, parotid gland, kidney, esophagus, stomach, pancreas	+
		breast	-
endodermal	urogenital system	prostate, exocervix, endocervix	+
		pro-endometrium (apical)	+ (single cells)
		sec-endometrium	-
		myometrium, ovary, liver	-
endodermal	respiratory ducts	lung	-
mesodermal	mesenchymal structures	skeletal muscles, fat tissue, soft tissue, heart	-
mesodermal	lymphatic tissue	lymph node, spleen, tonsils, thymus	-

+, positive staining; -, negative staining.
